# Verapamil inhibits early acute liver failure through suppressing the NLRP3 inflammasome pathway

**DOI:** 10.1111/jcmm.16357

**Published:** 2021-05-24

**Authors:** Mingying Han, Shouzhou Li, Lanrong Li

**Affiliations:** ^1^ Pediatric Intensive Care Unit Linyi People’s Hospital Linyi China; ^2^ Nutrition Department Chinese Medicine Hospital in Linyi City Linyi China; ^3^ Emergency Department Linyi People’s Hospital Linyi China

**Keywords:** acute liver failure, NLRP3 inflammasome, TXNIP, verapamil

## Abstract

Acute liver failure (ALF) is a rare disease characterized by the sudden onset of serious hepatic injury, as manifested by a profound liver dysfunction and hepatic encephalopathy in patients without prior liver disease. In this paper, we aim to investigate whether verapamil, an antagonist of TXNIP, inhibits early ALF through suppressing the NLRP3 inflammasome pathway. Firstly, an ALF mouse model was induced by lipopolysaccharide (LPS)/D‐galactosamine (GalN) treatment. The optimal concentration of verapamil in treating early ALF mice was determined followed by investigation on its mechanism in LPS/GalN‐induced liver injury. Western blot analysis and co‐immunoprecipitation were performed to determine the activation of the TXNIP/NLRP3 inflammasome pathway. Subsequently, overexpression of NLRP3 in mouse liver was induced by transfection with AAV‐NRLP3 in vivo and in vitro to identity whether verapamil inhibited early ALF through suppressing the activation of NLRP3 inflammasome. We found that ALF was induced by LPS/GalN in mice but was alleviated by verapamil through a mechanism that correlated with suppression of the NLRP3 inflammasome pathway. Oxidative stress and inflammatory response were induced by intraperitoneal injection of LPS/GalN, but alleviated with injection of verapamil. Overexpression of NLRP3 via AAV in mouse liver in vivo and in vitro reduced the therapeutic effect of verapamil on LPS/GalN‐induced ALF. Taken together, the TXNIP antagonist verapamil could inhibit activation of the NLRP3 inflammasome, inflammatory responses and oxidative stress to alleviate LPS/GalN‐induced ALF.

## 1 INTRODUCTION

Acute liver failure (ALF) is a challenging clinical syndrome characterized by massive death of hepatocytes and severe loss of liver function arising in eight weeks or less, and with ensuing hepatic encephalopathy.^1^, ^2^ Patients suffering from encephalopathy within 7 days of their liver failure have a good prognosis upon early recognition and transfer to a tertiary medical care centre with transplant facilities.^3^ However, the diverse inciting causes of ALFD such as drug‐evoked, metabolic, genetic, infectious, immune‐associated, haemodynamic and oncologic disorders make ALF a challenging disease.^4^, ^5^ Further investigation of the pathology of ALF may facilitate the discovery of new treatments or targets. In this regard, we note that there are two vital processes participating in the pathology of ALF. First, oxidative stress triggers a set of cysteine‐aspartate proteases known as caspases and induces overwhelming apoptosis of hepatocytes, and second, varying degrees of resultant inflammation are a major factor in inducing cerebral oedema and multiorgan system failure.^6^

Verapamil, a calcium channel blocker and P‐glycoprotein (P‐gp) blocker, has a definite therapeutic effect on ALF.^7^ Excessive calcium influx is one of important signals of cell death, and the level of the drug transporter P‐gp increased significantly in drug‐injured liver, while remaining little changed in other organs.^7^ Hence, verapamil can protect hepatocytes from dying in ALF. Meanwhile, verapamil is reported to have alleviated oxidative stress and exhibited hepatoprotective properties by reducing the level of the oxidation end‐product malondialdehyde (MDA).^8^ However, it still has not been clarified how verapamil mediates these latter effects.

It is reported that verapamil can inhibit the expression of thioredoxin‐interacting protein (TXNIP) and thus prevent islet cells from apoptosis.^9^ TXNIP can bind to thioredoxin 1 (TRX1), resulting in increased oxidative stress and inflammation, and can also bind to nucleotide binding oligomerization domain‐like receptors 3 (NLRP3), which promotes the release of IL‐1β and subsequent inflammation. During the treatment of chronic fatty hepatitis, verapamil plays a protective role through blocking the binding processes noted above.^10^, ^11^ Another study has revealed that verapamil can also treat acute hepatitis by up‐regulating anti‐inflammatory cytokine expression and down‐regulating pro‐inflammatory cytokines through inhibition of NF‐κB.^12^

The NLRP3 inflammasome is a cytosolic signalling complex related to the pathogenesis of many diseases and mediating the activation of inflammatory factors such as interleukin‐1 (IL‐1).^13^ The activity of the NLRP3 inflammasome can be perturbed by TXNIP silencing, which ultimately prevents inflammation and alters lipid metabolism.^14^ Given the therapeutic effect of verapamil on ALF and significant role of TXNIP/NLRP3 in inflammation and oxidative stress, we were eager to establish whether the inhibitory effect of verapamil on ALF is related to inhibition of NLRP3 inflammatory pathway and oxidative stress.

In this study, a mouse model of liver failure was induced by intraperitoneal injection of lipopolysaccharide and D‐galactosamine (LPS/GalN), which is a procedure often used to study the mechanism of clinical liver diseases and search for potential treatments.^15^ We next experimentally determined the optimal treatment dose of verapamil for ALF and investigated the mechanism by which verapamil functions to affect inflammatory and oxidative stress responses following ALF.

## 2 MATERIALS AND METHODS

### 2.1 Ethics statement

This research was approved by Animal Ethics Committee. The experiments involved animals were performed according to the ‘Regulation on the Administration of Laboratory Animals’ (State Scientific and Technological Commission of the People's Republic of China, 1 March 2017, revised edition) and guidelines for the care and use of experimental animals in the National Institutes of Health (ISBN: 13:978‐0‐309‐15400‐0, revised edition).

### 2.2 LPS/GalN‐induced ALF mice model

All the experimental animals fasted for 12 h before modelling. LPS (*E. coli* 055: b5) and GalN, purchased from Sigma‐Aldrich (Sigma‐Aldrich), were dissolved in phosphate‐buffered saline (PBS) to appropriate concentrations for an intraperitoneal injection volume of 0.1 mL. LPS (30 μg/kg) and GalN (600 mg/kg) ^16^ were intraperitoneally injected into mice to induce (ALF). After 30 minutes, verapamil 10 mg/kg (0.1 mL) (Fengzhulin Chemistry Technology Ltd) was intraperitoneally injected into the mice. The mice were anaesthetized with intraperitoneal injection with pentobarbital sodium (50 mg/kg) and fixed on the heating pad at 3 hours after inducing ALF. Then, the mice were anaesthetized with inhalation of methoxyflurane and killed by exsanguination. The liver and blood samples were collected and stored at –80℃ for subsequent experiments.

### 2.3 Experimental procedures and grouping

One hundred and eight C57BL/6 mice were purchased from Laboratory Animal Center of Hubei Disease Prevention and Control Center. 72 mice (12 mice for each group) were selected for survival analysis. The remaining 36 mice were randomly divided into 6 groups (6 mice per group), of which 5 groups received intraperitoneal (i.p) injection with LPS (30 μg/kg) and GalN (600 mg/kg) to induce ALF, followed by i.p. injection with different doses of verapamil (0, 5, 10 and 20 mg/ kg). The control group was injected with the same volume of saline.

Twenty‐four C57BL/6 mice were randomly divided into 4 groups (6 mice per group), and each group received i.p. injection with LPS (30 μg/kg) and GalN (600 mg/kg) to induce ALF. Mice in the LPS/GalN + verapamil group were intraperitoneally injected with verapamil (10 mg/kg) 30 minutes after inducing ALF. Mice in the verapamil along group were intraperitoneally injected with verapamil (10 mg/kg) without induction of ALF, and mice in the control group had no treatment. The mice were killed 3 hours after inducing ALF, and liver and blood samples were collected for subsequent experiments.

Twenty‐four C57BL/6 mice were randomly divided into 4 groups (6 mice per group) and were transfected with AAV‐NLRP3 (adeno‐associated virus construct encoding NLRP3) two weeks before ALF modelling. Mice transfected with AAV‐8 empty vector served as a control group. ALF was induced after the expression of NLRP3 had been stably induced. Mice in the LPS/GalN group were injected i.p. with LPS (30 μg/kg) and GalN (600 mg/kg) to induce ALF. Mice in the LPS/GalN + verapamil group were i.p. injected with verapamil (10 mg/kg) 30 minutes after inducing ALF, and mice in the verapamil group were i.p. injected with verapamil (10 mg/kg) without inducing ALF, whereas mice in the control group received no treatment. Mice were killed 3 hours after inducing ALF, and liver and blood samples were collected for subsequent experiments.

NCTC1469 hepatocytes were cultured in vitro and divided into control (no treatment), verapamil (hepatocytes treated with verapamil (10 mg/kg) without inducing LPS‐induced liver injury), LPS (hepatocytes treated with LPS (1 μg/mL) within the culture medium to simulate the LPS‐induced liver injury in vivo) and LPS + verapamil (hepatocytes treated with verapamil (10 mg/kg) 30 minutes after LPS‐induced liver injury) groups, as in the animal experiment. Then, the cells were transfected with AAV‐NLRP3 followed by the four groups of experiments as described above.

### 2.4 In *vitro* model of LPS‐induced liver injury

Murine liver cell NCTC1469, purchased from Bafeier Biotechnology Ltd, was cultured in Dulbecco's modified Eagle's medium (DMEM) with 10% (v/v) foetal bovine serum (FBS) in a humidified atmosphere with 5% CO_2_ at 37°C. LPS (1 μg/mL) was added into the medium when cell confluency reached 75% to establish the in vitro model of LPS‐induced liver injury. Six hours after induced injury, cells were collected for the subsequent experiments.

### 2.5 AAV‐NLRP3 construction and transfection

The AAV with encoding gene to overexpress NLRP3 was purchased from Genechem Ltd. The construction, recombination, amplification and purification of serotypes, specifically the transfection construction AAV‐8 and AAV‐NLRP3, were completed by Genechem Co., Ltd. AAV‐NLRP3 was injected via the portal vein or into the liver parenchyma of mice 2 weeks before induction of ALF to obtain stable transfection with AAV‐8.^17^ Mice injected with AAV‐8 empty vector served as control (AAV‐NC group) to verify the efficiency of NLRP3 overexpression.

NCTC1469 cells were seeded at 10^5^ cells per well in 6‐well plate. After 24 hours, cells were transfected with AAV‐NLRP3 and incubated for 4 h. Then, cells were washed with 10 mM PBS (pH = 7.4) with the culture medium renewed. Cells transfected with AAV‐8 empty vector served as control (AAV‐NC group) to verify the efficiency of NLRP3 overexpression. Cells were induced with injury by LPS as experimental groups.

### 2.6 Cell Counting Kit‐8 (CCK‐8) assay

The CCK‐8 kit was purchased from Beyotime Biotechnology and used following manufacturer's instruction. Portions of cell suspension (100 μL) were seeded in a 96‐well plate and incubated under 5% CO_2_ at 37°C for 24 hours. Afterwards, cells were transfected with AAV followed by LPS‐induced injury model establishment. After modelling, 10 µL CCK8 solution was added into each well for incubation for 1 to 4 hours. The absorbance was read by spectrometry at 450 nm and cell viability was calculated.

### 2.7 Annexin V apoptosis assay

Annexin V apoptosis assay was performed as follows. Falcon tubes were labelled according to the groups and washed with cold PBS twice. Then, cell suspension containing at 1 × 10^6^ cells/mL was prepared with 1 × Binding Buffer. Each Falcon tube was added with 100 μL cells suspension, and then gently mixed with Annexin V and dye according to the kit protocol. The cell apoptosis rate was determined with flow cytometer. All reagents were purchased from Bafeier Biotechnology Co., Ltd.

### 2.8 Terminal deoxynucleotidyl transferase dUTP nick end labelling (TUNEL) assay

The liver tissues were fixed with 4% paraformaldehyde, dehydrated and embedded in paraffin, and then cut into 4 μm sections for TUNEL assay. The sections were stained with a one‐step TUNEL apoptosis detection kit. First, the sections were treated with 20 mg/L protease K without DNase for 20 minutes and then incubated at 37°C for 1 hours with the mixture of the fluorescent labelling solution and terminal deoxynucleotidyl transferase (TdT) prepared as per instructions. After 1 × PBS washing, the sections were placed in the mounting reagent containing DAPI to stain the nucleus. The fluorescent image was captured with an inverted fluorescence microscope (model: TH4‐200, Olympus Corporation) within six randomly selected views for apoptosis assessment. The apoptosis rate was calculated according to formula: Apoptosis rate = TUNE‐positive cells / total cells × 100%.

### 2.9 Haematoxylin and eosin (HE) staining and histological score

The paraffin‐embedded liver tissue was cut into 4 μm‐thick sections for histological analysis by HE staining. The cross‐section images were captured by a Leica Microsystems microscope (model: DM2000, CMS GmbH). Six views of each section were randomly selected to evaluate the liver injury. According to the standard of Suzuki,^18^ three representative indexes of liver injury (congestion, vacuolation and necrosis) were scored from 0 to 4 according to their severity (0 for none; 1 for extremely mild; 2 for mild; 3 for moderate; and 4 for severe), thus to a maximum possible score of 12.

### 2.10 Measurement of MDA, reactive oxygen species (ROS) and inflammatory cytokines

The liver tissue preserved in −80°C was homogenized with saline to prepare 10% w/v liver tissue homogenate (each 1 μg liver tissue was suspended in 9 μL saline). A bicinchoninc acid (BCA) kit was used to determine the protein concentration in the homogenate. Afterwards, the sample was centrifuged for ten min at 14 000 *g* and the supernatant was collected. Then, the MDA and ROS contents in liver tissue were determined using corresponding kits purchased from Jiancheng Bioengineering Institute Ltd. TNF‐α, IL‐6, IL‐1β and IL‐18 in liver tissue were detected using ELISA kits (Elabscience Biotechnology Co. Ltd) according to the manufacturer's protocol.

### 2.11 Measurement of alanine aminotransferase (ALT) and aspartate transaminase (AST) in serum

Fresh blood (5 mL) was collected from mice and centrifuged at 1000 *g* to collect the serum. The ALT and AST levels released from liver tissue were measured using an AU5800 series automatic biochemical analysis system (Beckman Coulter Laboratory Systems Co Ltd) and ALT/AST‐specific detection kits (Beckman Coulter Laboratory Systems). The result was used to determine the injury on hepatocytes.

### 2.12 Immunohistochemistry (IHC)

The liver tissue was fixed with 4% paraformaldehyde and embedded in paraffin, then cut into 4 μm‐thick sections. The section was used for IHC staining following standard procedures. Then, the sections were incubated with the primary antibody of caspase‐1 (1:200, 22915‐1‐AP, ProteinTech) followed by additional incubation with HRP‐labelled secondary antibody and colour development in DAB to observe the binding of the antibodies, with haematoxylin counterstaining. Cross‐sectional images were captured by a microscope (Leica Microsystems model DM2000). Six views of each section were randomly selected by ImagePro Plus 6.0 (Media Cybernetics Inc) for analysis of cumulative immunohistochemical optical density (IOD).

### 2.13 Enzyme‐linked immunosorbent assay (ELISA)

The ELISA kit (Jiangsu Enzyme Labeling Company) was taken from the refrigerator 1 hours in advance to warm to room temperature. Then, the standard well and the sample well were set up, and 50 μL of the standard and 50 μL of the corresponding sample were added to the standard well and the sample well, respectively. A total of 100 μL of enzyme‐labelled solution was added to all wells and incubated at 37°C for 60 minutes, and the plate was washed with washing solution for 4 times. Then, 300 μL of washing solution was added to each well followed by removal of the liquid after 30 seconds, and the plate was washed 5 times. Next, 100 μL of enzyme conjugate working solution was added to each well except the blank well. The reaction wells were sealed and incubated at 37°C for 30 minutes. Afterwards, 50 μL of substrate A and B solutions was added to each well and incubated at 37°C for 15 minutes in the dark. Finally, 50 μL of stop solution was added to each well and the OD value was measured immediately after mixing.

### 2.14 Reverse transcription‐quantitative polymerase chain reaction (RT‐qPCR)

The total RNA was extracted from the liver tissue preserved at −80℃ with Trizol reagent, followed by quantitation by Nano drop (Thermo Fisher Scientific Inc). Oligo (dT) primers and reverse transcriptase (Thermo Fisher Scientific Inc) were used to reverse transcribe the total RNA (1 μg) into cDNA. The expression of relative mRNA was analysed by the 2^‐∆∆^Ct method, with GAPDH as the internal reference. Primers for GAPDH were (forward) 5'‐GCTAACATCAAATGGGGTG‐3' and (reverse) 5'‐TTGCTGACAATCTTGAGGGAG‐3'. PCR was performed using StepOnePlus (Applied Biosystems). The reaction proceeded for 1 cycle at 95°C for 15 minutes and 40 cycles at 95°C for 10 seconds and 60°C for 60 seconds.

### 2.15 Western blot

The total protein was extracted from liver tissue samples using RIPA lysis buffer (the ratio of liver tissue to RIPA lysis buffer was 50 mg: 1 mL). The nuclear and cytoplasmic proteins were extracted by using the nuclear and cytoplasmic protein extraction kit (Beyotime Biotechnology). The protein concentration of the extracted homogenate was determined by a BCA kit. Then, 5 × loading buffer (reducing) was used to prepare the protein sample for SDS‐polyacrylamide gel electrophoresis (PAGE). The protein was transferred to PVDF membrane (0.45 μm) by wet transfer. Then, the PVDF membrane was incubated with the primary antibodies overnight as follows: active caspase‐3 (1:500, bsm‐33199 M, Bioss), caspase‐3 precursor (1:500, bs‐2593R, Bioss), ASC (1:500, bs‐6741R, Bioss), IL‐1β (1:1000, bs‐20449R, Bioss), Bax (1:1000, 50599‐2‐Ig, ProteinTech Group, Inc Chicago, USA), Bcl2 (1:1000, 12789‐1‐AP, ProteinTech Group), TXNIP (1:1000, 18243‐1‐AP, ProteinTech Group), caspase‐1 (1:1000, 22915‐1‐AP, ProteinTech Group) and IL‐18 (1:1000, 10663, ProteinTech Group). Then, the membrane was incubated with HRP‐labelled goat anti‐rabbit or goat anti‐mouse secondary antibodies at room temperature for 1 hours. The protein expression was detected with ECL luminescent solution. β‐actin was used as internal reference of total protein, and histone‐H3 was used as the internal reference of nuclear protein. The quantitative analysis of protein bands was analysed by Image J software (NIH).

### 2.16 Co‐immunoprecipitation (Co‐IP)

The liver tissue stored at −80°C was homogenized in RIPA lysis buffer (the ratio of liver tissue and RIPA lysis buffer was 50 mg: 1 mL), and the protein concentration of the extracted homogenate was determined by a BCA kit. According to the protein concentration, total protein lysate (1 mg) was used for Co‐IP. The lysate was incubated with antibody (1 μg) against TXNIP or rabbit polyclonal IgG antibody as control. The mixture was shaken at 4°C for 4 hours to allow full binding to the corresponding antigens. Then, 20 μL re‐suspended Protein A/G PLUS‐agarose was added to the samples and rotated for the 2 hours to ensure full adsorption of the antibody‐antigen complex by Protein A/G PLUS‐agarose. Then, 5 × loading buffer (reducing) was used to prepare the protein sample solution for Western blot analysis, much as described above. The antibodies included ASC (1:50, ba‐6741R), TXNIP (1:100, 18243‐1‐AP), Trx (1:50, 14999‐1‐AP) and NLRP3 (1:50, 19771‐1‐AP). The binding of the proteins was determined by quantitative analysis using image J software (NIH).

### 2.17 Statistical analysis

Data are analysed using SPSS 21.0 software (IBM) and expressed as mean ± standard deviation. Unless otherwise noted, statistical comparisons were performed using an unpaired t test when only two groups were compared, or by Tukey's test‐corrected one‐way analysis of variance (ANOVA) with when more than two groups were compared. The Kaplan‐Meier method was used to calculate the survival rate of mice, and log‐rank test was used for univariate analysis. *P* <.05 indicates statistical significance.

## 3 RESULTS

### 3.1 The optimal concentration of verapamil in treating early ALF

**FIGURE 1:**
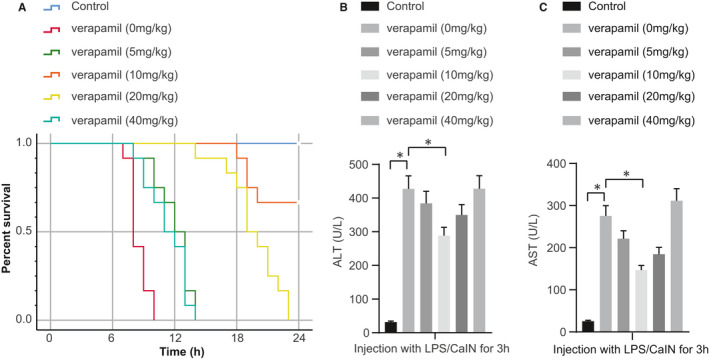
The selection of the optimal concentration of verapamil in treating early ALF. A, Survival rate of mice in each group; B, ALT levels in mice serum in each group detected with assay kits; C, AST levels in mice serum in each group detected with assay kits; *P <.05. All values are expressed as mean ± standard deviation. Statistical comparisons are performed by Tukey's test‐corrected one‐way analysis of variance (ANOVA) when more than two groups were compared. The survival rate of mice is calculated by Kaplan‐Meier method, n = 6

First, we established the optimal dose of verapamil in treating early ALF. LPS/GalN‐induced ALF model is a mature technique used for studying the mechanisms of clinical liver disease and potential treatments.^15^ The ALF model was induced in C57BL/6 mice by LPS/GalN treatment. After 30 minutes, mice were treated with various doses of verapamil and then observed to examine the survival rate of each group. The longest survival time of the LPS/GalN‐induced mice without verapamil treatment was only 10 hours, while the application of verapamil can significantly prolong the survival time of LPS/GalN‐induced mice, and verapamil at a dose of 10 mg/kg showed the most significant effect on prolonging the survival time (Figure 1A). After observation of the survival time of the mice in each group, 36 mice were selected for LPS/GalN‐induced modelling again and the levels of ALT and AST in serum were measured to evaluate the ALF after modelling. Due to survival time of ALF mice being as little as 7 hours, the serum levels of ALT and AST were detected 3 hours after modelling. The results showed that the ALT and AST levels in serum were significantly increased at 3 hours after induction of ALF in mice. However, the application of verapamil after modelling could significantly inhibit the ALT and AST levels, with 10 mg/kg showing the most significant inhibitory effect (Figure 1A,B). Therefore, this dose was chosen in the following research.

### 3.2 Verapamil alleviates LPS/GalN‐induced ALF

**FIGURE 2:**
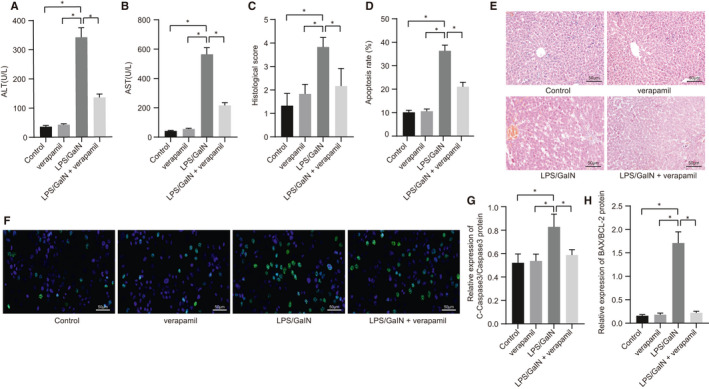
Verapamil alleviates LPS/GalN‐induced ALF. Mice were induced by LPS/GalN or treated with verapamil alone or in combination. A, ALT levels in mice serum in each group detected with assay kit; B, AST levels in mice serum in each group detected with assay kit; C, the condition of ALF of mice in each group scored according to standard scores; D, the apoptosis rate of mice in each group; E, the morphology of liver tissue observed using HE staining (scale bar: 50 um); F, the apoptosis rate of hepatocytes in each group assessed using TUNEL staining (scale bar: 50 um); G, statistics of relative expression of C‐caspase‐3/caspase‐3 protein; H, statistics of relative expression of BAX/BCL‐2 protein. *P <.05. All values are expressed as Mean ± standard deviation. Statistical comparisons are performed by Tukey's test‐corrected one‐way analysis of variance when more than two groups were compared, n = 6

After confirming the optimal concentration of verapamil for ALF, we tried to further clarify the therapeutic effect of verapamil on ALF. It was evident that, compared with the control and verapamil groups, serum levels of ALT and AST were significantly increased in the LPS/GalN group (Figure 2A,B) and histological score was significantly increased (Figure 2C), but was reduced by verapamil treatment. As reflected by TUNEL and HE staining, apoptosis, congestion, vacuolation and necrosis were significantly increased in liver of the LPS/GalN group, which could be reduced after addition of verapamil (Figure 2D‐F). In addition, the expression of C‐caspase‐3/caspase‐3 and BAX/BCL‐2 was significantly increased in the LPS/GalN group, which could be decreased after addition of verapamil (Figure 2G,H). In conclusion, verapamil inhibits LPS/GalN‐induced ALF.

### 3.3 Verapamil alleviates LPS/GalN‐induced inflammatory response and oxidative stress

**FIGURE 3:**
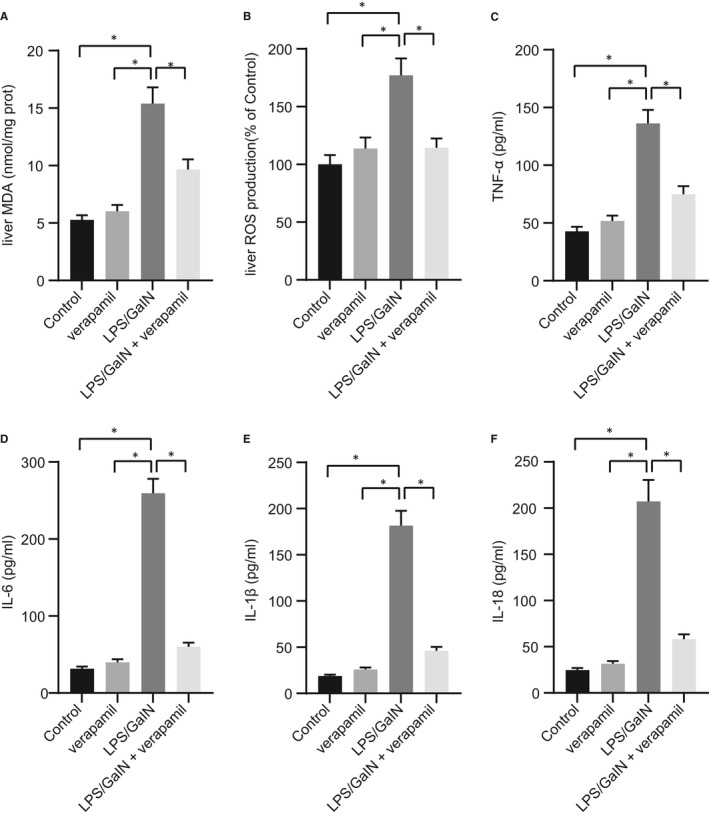
Verapamil represses LPS/GalN‐induced inflammatory response and oxidative stress. Mice were induced by LPS/GalN or treated with verapamil alone or in combination. A, MDA in mice liver tissue detected with specific kits; B, ROS levels in mice liver tissue detected with specific kits; C, TNF‐α levels in homogenate of mice liver tissue detected with ELISA kit. D, IL‐6 levels in homogenate of mice liver tissue detected with ELISA kit; E, IL‐1β levels in homogenate of mice liver tissue detected with ELISA kit; F, IL‐18 levels in homogenate of mice liver tissue detected with ELISA kit. *P <.05. All values are expressed as mean ± standard deviation. Statistical comparisons are performed by Tukey's test‐corrected one‐way analysis of variance when more than two groups were compared, n = 6

Here, we explored further the alleviation by verapamil on the inflammatory response and oxidative stress in LPS/GalN‐induced ALF, with the determination of plasma concentration of TNF‐α and IL‐6 (the representative inflammatory cytokines), IL‐1β and IL‐18 (the cytokines down‐stream of the TXNIP/NLRP3 inflammasome pathway) as well as the oxidative stress markers MDA and ROS. We found that intraperitoneal injection of LPS/GalN significantly induced oxidative stress and inflammatory response, as evidenced by elevated levels of MDA, ROS, TNF‐α, IL‐6, IL‐1β and IL‐18, but the injection of verapamil in modelled mice alleviated the liver inflammatory response and oxidative stress (Figure 3A‐F). Thus, verapamil postponed or attenuated the LPS/GalN‐induced inflammatory response and oxidative stress.

### 3.4 Verapamil blocks LPS/GalN‐induced activation in TXNIP/NLRP3 inflammasome pathway

**FIGURE 4:**
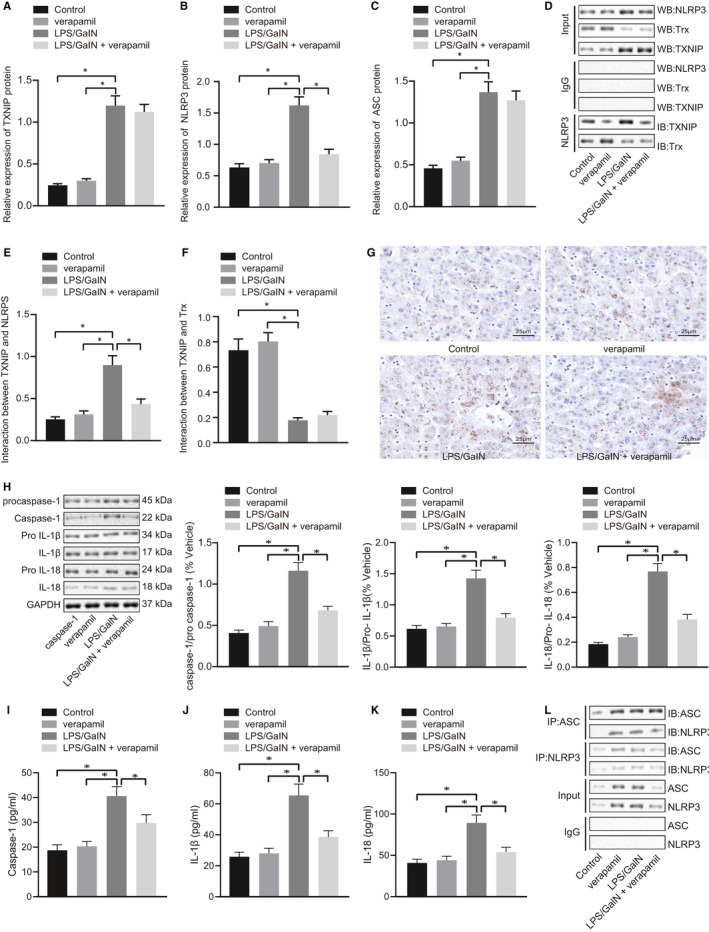
Verapamil inhibits LPS/GalN‐induced activation of the TXNP/NLRP3 inflammasome pathway. Mice were induced by LPS/GalN or treated with verapamil alone or in combination. A, The protein expression of TXNP determined with Western blot; B, the protein expression of NLRP3 determined with Western blot; C, the protein expression of ASC determined with Western blot; D, the binding of TXNP and NLRP3, TXNO and Trx determined with Co‐IP; E, the interaction between TXNP and NLRP3 analysed by IHC; F, the interaction between TXNO and NLRPS analysed by IHC; G, The expression of caspase‐1 detected with IHC (scale bar: 25 um); H, the expression of pro‐caspase‐1/caspase‐1, pro‐IL‐1β/IL‐1β and pro‐IL‐18/IL‐18 determined with Western blot. I, The expression of enzyme activity of caspase‐1 in mice liver tissue detected by ELISA; J, the expression of IL‐1β in mice liver tissue detected by ELISA. K, The expression of IL‐18 in mice liver tissue detected by ELISA. L, The interaction between NLRP3 and ASC. *P <.05. All values are expressed as mean ± standard deviation. Statistical comparisons are performed by Tukey's test‐corrected one‐way analysis of variance when more than two groups were compared, n = 6

We next explored further whether the rescue by verapamil of LPS/GalN‐induced ALF was correlated with effects on the TXNIP/NLRP3 inflammasome pathway. First, the expression of TXNIP, NLRP3 and ASC, the key proteins in inflammasome pathway, was determined in mouse liver in each group. The results of Western blot analysis showed that the expression of TXNIP, NLRP3 and ASC were significantly higher in LPS/GalN group than in the control and verapamil groups. Verapamil inhibited the expression of NLRP3 but had no effect on the expression of TXNIP and ASC after intraperitoneal injection of LPS/GalN (Figure 4A‐C). Previous studies have shown that TXNIP inactively binds to Trx under physiological conditions, while TXNIP dissociates from Trx and binds to NLRP3 under oxidative stress, thereby activating the down‐stream inflammatory pathway of NLRP3.^19^ Therefore, the binding between TXNIP and NLRP3, TXNIP and Trx in mice liver of each group was further investigated using Co‐IP. The results demonstrated that the formation of TXNIP/NLRP3 complex was increased and TXNIP/Trx complex was decreased after injection of LPS/GalN, but that verapamil treatment significantly inhibited the formation of the TXNIP/NLRP3 complex after intraperitoneal injection of LPS/GalN, while having no effect on the TXNIP/Trx complex (Figure 4D‐F).

The activation of NLRP3 leads to its oligomerization, whereupon NLRP3, ASC and pro‐caspase‐1 are assembled into the inflammasome complex, which triggers the transformation of pro‐caspase‐1 to caspase‐1, as well as the production and secretion of mature IL‐1β and IL‐18.^20^ In the following experiments, we measured the expression of proteins and cytokines in the down‐stream of TXNIP/NLRP3 inflammasome pathway, including caspase‐1, IL‐1 and IL‐18. The results in IHC and Western blot assays revealed that the expression of caspase‐1 protein in the LPS/GalN group increased significantly compared with the control and verapamil groups. Verapamil robustly inhibited caspase‐1 expression after intraperitoneal injection of LPS/GalN (Figure 4G‐I). Western blot results showed that the protein expression of caspase‐1, IL‐1β and IL‐18 in the LPS/GalN group was significantly higher than that in the control and verapamil groups. Besides, verapamil treatment significantly inhibited the expression of caspase‐1, IL‐1β and IL‐18 after intraperitoneal injection of LPS/GalN (Figure 4H‐K). Co‐IP results presented that the binding level of NLRP3 to ASC was significantly increased in the LPS/GalN group compared with the control group and the verapamil group and that application of verapamil after intraperitoneal injection of LPS/GalN could significantly inhibit the binding level of NLRP3 to ASC (Figure 4L). Altogether, verapamil exerted an inhibitory effect on LPS/GalN‐induced activation in TXNIP/NLRP3 inflammasome pathway.

### 3.5 Overexpression of NLRP3 eliminates therapeutical effect of verapamil on ALF

As indicated by the above‐mentioned findings, NLRP3 may play a critical role in verapamil‐induced alleviation on ALF. In the following study, we explored further whether verapamil regulated the TXNIP/NLRP3 inflammasome pathway to alleviate ALF through overexpression of NLRP3 obtained by AAV‐NLRP3 treatment in mice.

**FIGURE 5:**
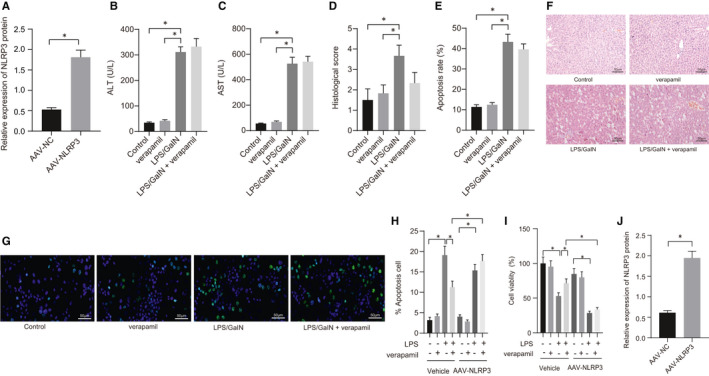
Overexpression of NLRP3 eliminates verapamil‐induced effect on ALF. Mice or cells were induced by LPS/GalN or treated with verapamil alone or in combination. A, The expression of NLRP3 protein in AAV‐NC and AAV‐NLRP3 groups determined by Western blot; B, ALT levels in mice serum in each group detected with assay kits; C, AST levels in mice serum in each group was detected with assay kits; D, the condition of ALF of mice in each group scored according to standard evaluation; E, the apoptosis rate of cells in mice of each group; F, the morphology of liver tissue observed using HE staining (scale bar: 50 um); G, the apoptosis rate of hepatocytes in each group assessed using TUNEL staining (scale bar: 50 um). * indicates *P* <.05 of LPS/GalN group compared with control or verapamil groups. ^#^indicates *P* <.05 of LPS/GalN + verapamil group compared with LPS/GalN group. H, The apoptosis of mouse hepatocytes examined with flow cytometry; I, the cell viability of murine hepatocytes was determined with CCK8 kit; J, the expression of NLRP3 protein in AAV‐NC and AAV‐NLRP3 groups determined by Western blot. *P <.05. All values are expressed as mean ± standard deviation. Statistical comparisons are performed by Tukey's test‐corrected one‐way analysis of variance (ANOVA) when more than two groups were compared, n = 6

First, the efficiency of NLRP3 overexpression using AAV via portal vein injection was verified by Western blot, which showed that the expression of NLRP3 protein was significantly promoted after portal vein injection of AAV‐NLRP3 in mice, compared with the AAV‐NC group (Figure 5A). We next investigated whether the overexpression of NLRP3 modulated the effect of verapamil on ALF. We found that, compared with the control and verapamil groups, ALT and AST levels, as well as histological scores, were increased significantly in the LPS/GalN group (Figure 5B‐D). TUNEL staining showed that apoptosis in the LPS/GalN group was conspicuously increased (Figure 5E‐G). However, verapamil did not reduce liver injury after overexpression of NLRP3, as evidenced by the lack of significant improvement in histology scores (Figure 5F) and no significant decrease in apoptosis (Figure 5G).

Moreover, in vitro experiments were performed to further verify the therapeutic effect of verapamil on ALF. We found that LPS significantly induced apoptosis and reduced the viability of hepatocytes in vitro, which was reversed by verapamil. However, the overexpression of NLRP3 by AAV‐NLRP3 in murine hepatocytes significantly eliminated the protective effect of verapamil (Figure 5H‐J).

**FIGURE 6:**
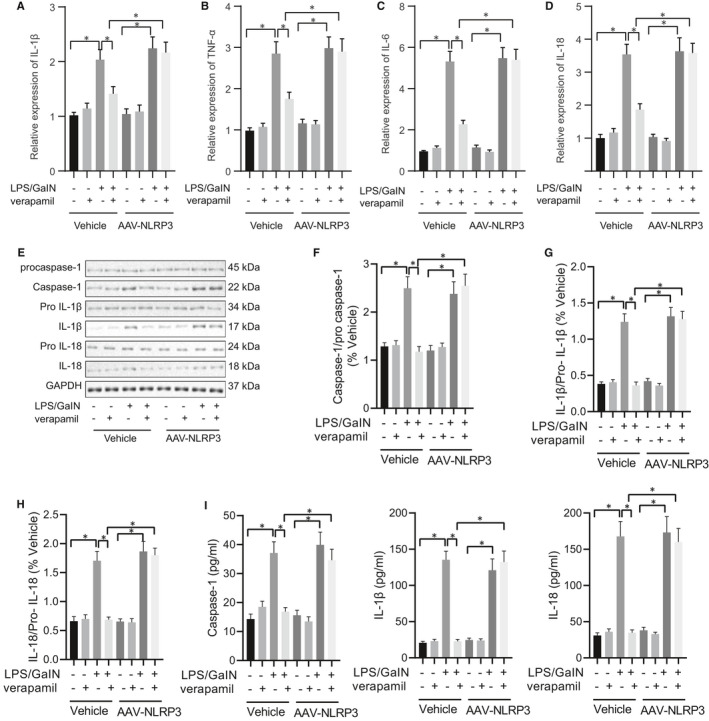
Overexpression of NLRP3 eliminates the verapamil‐induced inhibitory effect on the TXNP/NLRP3 inflammasome pathway. Mice were induced by LPS/GalN or treated with verapamil. A, The mRNA levels of IL‐1β in mouse liver tissue in each group determined with RT‐qPCR; B, the mRNA levels of TNF‐α in mouse liver tissue in each group determined with RT‐qPCR; C, the mRNA levels of IL‐6 in mouse liver tissue in each group determined with RT‐qPCR; D, the mRNA levels of IL‐18 in mouse liver tissue in each group determined with RT‐qPCR; E, the expression of pro‐caspase‐1/caspase‐1, pro‐IL‐1β/IL‐1β and pro‐IL‐18/IL‐18 proteins in mouse liver tissue in each groups determined with Western blot; F, the relative expression of caspase‐1 normalized to GAPDH. G, the relative expression of IL‐1β normalized to GAPDH; H, the relative expression of IL‐18 normalized to GAPDH; I, the activity of caspase‐1, IL‐1β and IL‐18 measured by ELISA. *P <.05. All values are expressed as mean ± standard deviation. Statistical comparisons are performed by Tukey's test‐corrected one‐way analysis of variance (ANOVA) when more than two groups were compared, n = 6

Finally, via overexpression of NLRP3, we confirmed that verapamil treated ALF through inhibiting the TXNIP/NLRP3 inflammasome pathway. It was evident that after overexpression of NLRP3 using AAV‐NLRP3 in mice, LPS/GalN could still induce ALF, in which IL‐1β, TNF‐α, IL‐6 and IL‐18 levels were increased. However, with NLRP3 overexpression, verapamil did not significantly inhibit the expression of these inflammatory cytokines after LPS/GalN modelling (Figure 6A‐D). Additionally, after overexpression of NLRP3 in mice, LPS/GalN significantly induced the expression of caspase‐1, IL‐1β and IL‐18, which could not be inhibited by verapamil (Figure 6E‐6I). The results suggested that NLRP3 played a critical role in the treatment of ALF with verapamil.

## 4 DISCUSSION

**FIGURE 7:**
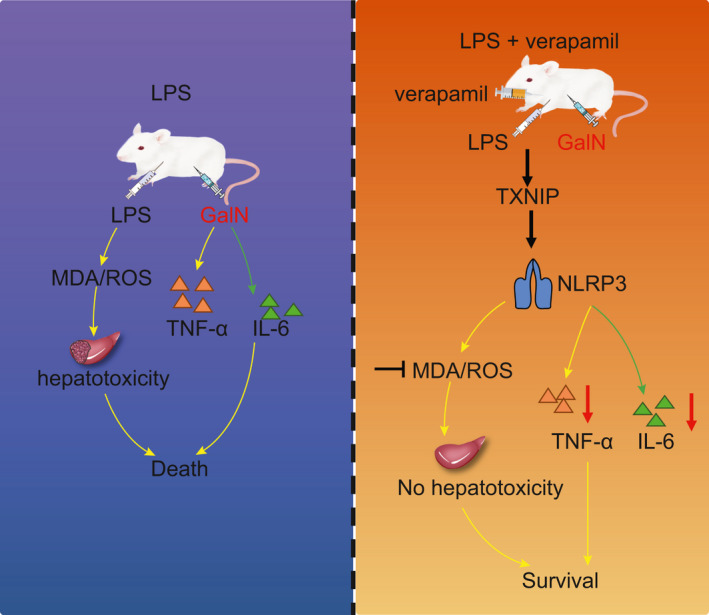
Schematic map referring to the role of TXNIP antagonist verapamil in ALF. TXNIP antagonist verapamil could inhibit activation of the NLRP3 inflammasome, inflammatory responses, and oxidative stress to alleviate LPS/GalN‐induced ALF

Verapamil is a commonly used calcium channel blocker and P‐glycoprotein inhibitor, which can play therapeutic roles in cardiovascular diseases,^21^, ^22^, ^23^ diabetes ^11^, ^24^, ^25^ and certain liver diseases including liver fibrosis,^26^ liver ischaemia‐reperfusion injury ^27^ and hepatic metaflammation.^10^ Based on its protective effects, verapamil may treat liver disease by inhibiting oxidative stress and inflammatory response, which is the early pathological manifestation of ALF. In addition, Yumoto et al proved that ALF can be treated or arrested with verapamil.^7^ We first identified the optimal dose of verapamil (10 mg/kg) for the treatment of early ALF, which was not hither addressed. Strikingly, we then identified the mechanism that verapamil can reduce TXNIP/NLRP3 inflammatory pathway, so as to reduce oxidative stress and inflammatory responses to alleviate early ALF (Figure 7).

In the subsequent experiments, we developed the LPS/GalN‐induced ALF mouse model and demonstrated that verapamil could alleviate LPS/GalN‐induced ALF as manifested by significant decrease of serum ALT and AST levels and rescued cell apoptosis in the liver tissues. LPS is the main bacterial component that can trigger the activation of innate immune cells and provoke an inflammatory response. GalN, a kind of hepatotoxin that inhibits protein and RNA synthesis, can lead to fatal acute liver failure when administered in combination with LPS.^16^ After being treated by LPS, macrophages will release pro‐inflammatory cytokines and produce reactive oxygen species (ROS). Of note, inflammation and oxidative stress are the key features in the early stage of liver failure.^28^ NLRP3 inflammasome activation induced by accumulation of ROS‐damaged mitochondria can stimulate an inflammatory response.^29^ The consequently increasing activity of caspase‐1, a component of the NLRP3 inflammasome, leads to secretion of inflammatory factors (IL‐1β etc).^30^ After verapamil treatment, the levels of oxidative stress indicators such as MDA, ROS and the release of inflammatory proteins including TNF‐α, IL‐6, IL‐1β and IL‐18 decreased dramatically in mode mice, thus demonstrating the inhibitory effects of verapamil on inflammation and oxidative stress.

Moreover, we unravelled that administration of verapamil could down‐regulate NLRP3 in mice with LPS/GalN‐induced ALF and then repress the formation of inflammasomes through impairing the TXNIP/NLRP3 complex. The NLRP3 inflammasome, a complex consisting of NLRP3 protein, ASC and pro‐caspase‐1, can trigger the generation of caspase‐1 and secretion of mature IL‐1β and IL‐18.^31^ Hence, down‐regulation of NLRP3 may reduce the release of pro‐inflammatory cytokines that contributes to the suppression of inflammation. A recent study has also indicated that down‐regulating the NLRP3 inflammasome activity in macrophages can alleviate symptoms of ALF.^32^ Moreover, it has been elsewhere demonstrated that NLRP3 inflammasome activation could be impaired by TXNIP deficiency.^33^ TXNIP, an endogenous inhibitor of TRX, is reported to induce acute ischaemic stroke through inflammasome activation and redox imbalance.^34^ Besides, exosomal miR‐17 could target TXNIP and inhibit the activation of inflammasome in liver macrophages to treat ALF.^35^ Additionally, verapamil can treat non‐alcoholic fatty liver disease by inhibiting the TXNIP/NLRP3 pathway and reducing the level of IL‐1β and IL‐18 to attenuate hepatic metaflammation.^10^ Consistent with these results, the restoration of NLRP3 counteracted the protective effect of verapamil on hepatocytes against ALF‐induced injury. Our data further evidenced that verapamil exerted a hepatoprotective effect through impairing the binding of TXNIP to NLRP3. Similarly, inhibition of TXNIP/NLRP3 inflammasome activation by curcumin protects against liver inflammation, hence suggesting a hepatoprotective effect of curcumin ^36^ whereas the amino acid taurine attenuates Schistosoma‐induced liver injury by disrupting the TXNIP/NLRP3 pathway.^37^ Another study has revealed that overexpression of TXNIP and NLRP3 is associated with the down‐regulated antioxidant genes such as catalase and MnSOD,^38^ which was consistent with an anti‐oxidative effect of verapamil reported in this study.

In conclusion, we identified the optimum verapamil dose (10 mg/kg) for treating the ALF mouse model and showed that verapamil can alleviate early ALF by inhibiting the TXNIP/NLRP3 pathway, which was otherwise associated with inflammation and oxidative stress. The relationship among the NLRP3 pathway, inflammatory responses and oxidative stress is complex. Although we have not fully articulated this complex mechanism, our findings have laid a foundation for the more appropriate application of verapamil for liver disease in clinical practice.

## CONFLICT OF INTEREST

The authors confirm that there are no conflicts of interest.

## AUTHOR CONTRIBUTION

**Mingying Han:** Conceptualization (equal); Data curation (lead); Formal analysis (lead); Project administration (lead); Validation (lead); Writing‐original draft (equal). **Shouzhou Li:** Conceptualization (equal); Software (lead); Visualization (lead); Writing‐original draft (equal). **Lanrong Li:** Conceptualization (equal); Investigation (lead); Methodology (lead); Resources (lead); Supervision (lead); Writing‐review & editing (lead).

## Data Availability

The datasets generated during the current study are available.
